# How to not revert to type: Complexity-informed learnings from the pandemic response for health system reform and universal access to integrated care

**DOI:** 10.3389/fpubh.2023.1088728

**Published:** 2023-02-17

**Authors:** Sarah Parker, Luisne Mac Conghail, Rikke Siersbaek, Sara Burke

**Affiliations:** Centre for Health Policy and Management, School of Medicine, Trinity College Dublin, Dublin, Ireland

**Keywords:** universal healthcare, integrated care, complexity theory, health system reform, COVID-19, Ireland, complexity science, systems thinking

## Abstract

This article is part of the Research Topic ‘Health Systems Recovery in the Context of COVID-19 and Protracted Conflict’.

**Background:**

COVID-19 has highlighted existing health inequalities and health system deficiencies both in Ireland and internationally; however, understanding of the critical opportunities for health system change that have arisen during the pandemic is still emerging and largely descriptive. This research is situated in the Irish health reform context of Sláintecare, the reform programme which aims to deliver universal healthcare by strengthening public health, primary and community healthcare functions as well as tackling system and societal health inequities.

**Aims and objectives:**

This study set out to advance understanding of how and to what extent COVID-19 has highlighted opportunities for change that enabled better access to universal, integrated care in Ireland, with a view to informing universal health system reform and implementation.

**Methods:**

The study, which is qualitative, was underpinned by a co-production approach with Irish health system leadership. Semi-structured interviews were conducted with sixteen health system professionals (including managers and frontline workers) from a range of responses to explore their experiences and interpretations of social processes of change that enabled (or hindered) better access to universal integrated care during the pandemic. A complexity-informed approach was mobilized to theorize the processes that impacted on access to universal, integrated care in Ireland in the COVID-19 context.

**Findings:**

A range of circumstances, strategies and mechanisms that created favorable system conditions in which new integrated care trajectories emerged during the crisis. Three key learnings from the pandemic response are presented: (1) nurturing whole-system thinking through a clear, common goal and shared information base; (2) harnessing, sharing and supporting innovation; and (3) prioritizing trust and relationship-building in a social, human-centered health system. Policy and practice implications for health reform are discussed.

## Introduction

“*An understanding of change in the health field enables us to imagine and design alternative paths to the future*” [(1), p. 20].

Health system reform is a planned and purposeful process that involves attempts to (re)organize healthcare in a way that promotes the goals of equity, effectiveness, and efficiency (2). As Frenk [(1), p. 19] states, it is often initiated in response to the complexities posed when “nations are facing the simultaneous burdens of old, unresolved problems and new, emerging challenges”. While different forces and contexts have prompted system-level changes in health over the last decade, one such challenge that has reoriented a sense of urgency toward addressing poorly functioning healthcare is COVID-19. At the same time, this renewed focus on health system deficiencies has also created opportunities for reflection, learning and change (3), with evidence suggesting that the pandemic has accelerated reform of “long-standing structural weaknesses and priorities” that may have previously lacked political will or funding [(4), p. 2].

This is demonstrated across OECD countries by notable shifts in care delivery models toward telehealth/telemedicine as well as more flexible funding and staffing models; however, perhaps most significant has been the prioritization of non-acute (community) care to better serve patients outside of hospitals, help maintain access to routine care and minimize spread of the virus (5). The goal of hospital avoidance via the linking of acute and community services arguably “reflects the interconnected nature” of health systems and underscores the importance of bolstering community capacity in the COVID-19 context [(4), p. 2]. Yet the aim of shifting left, where prevention and integration are key and delivery in community settings is preferable, has remained a challenge in many jurisdictions, often despite long-standing policy intent (6).

This is particularly the case in Ireland, where current government policy aims to progress a reform agenda to transition to a health system based solely on need rather than ability to pay and a reorientation of the system toward providing care in the most appropriate setting (7, 8). Ireland remains one of the few high-income countries where citizens do not have universal access to public healthcare; rather, a complex set of eligibility arrangements based on age, health and socioeconomic status continue to be in place, many of which have been critiqued as antiquated and not fit-for-purpose. Just under half of the population purchases voluntary health insurance for access to private health services, which are generally oriented toward elective acute hospital-based care.

A core goal of the 10-year reform roadmap currently being implemented - called *Sláintecare*, Sláinte being the Irish word for health - is to deliver universal healthcare by strengthening public health, primary and community healthcare functions while also tackling health inequities. Within this remit is the planned development of integrated care pathways, where care is delivered “at the lowest level of complexity whether at home, near home, in hospital or via integrated care structures” [(7), p. 23]. Some progress has been made in this area (9); however, critical understanding of how the pandemic response could better-inform improved access to universal integrated care is still emerging and largely undeveloped. Access to universal integrated care is a policy goal in many health systems in high-income countries, including those in the UK, Ireland, New Zealand and numerous European regions (see, for example, (10, 11)).

Researching complex coordinated care models of this kind requires a whole-of-system approach [(12), p. 1]. System approaches acknowledge the interdependencies between health system levels and components, and recognize that the extent to which they are integrated or not will impact overall effectiveness (3). Incorporating understanding of the relationships between the organizations and agents comprising a health system, their interactions with the external environment and their ability to adapt to constantly evolving context(s), is therefore essential to guide health system change (1, 12, 13). Failure to do so can result in “silos of care”, where little attention is paid to “the patient transitions and communication channels between them” [(14), p. 2].

It can be said that the success of COVID-19 responses largely – though not always - depended on how existing health systems were “organized, governed and financed across all levels in a coordinated manner” [(15), p. 964]. For this reason, there is a need to better understand and learn from the interconnected elements of national pandemic responses through a complexity (or complex systems) lens. Using Ireland as a case study, we mobilize a complexity-informed approach to generate research evidence that enables lesson drawing (16) to guide universal health reform, with a view to facilitating better access to universal integrated care in the COVID-19 context.

The aim of this paper is to contribute to the emerging academic discussion on how key learnings from health systems' pandemic responses can be used to inform health system change. Presenting data from a qualitative study of the Irish health system response to COVID-19, this research demonstrates the value of applying complexity to: (1) create a more nuanced, explanatory account of the processes that impacted on access to universal integrated care during the pandemic; and (2) generate policy and practice recommendations that seek to ensure solutions that emerged during COVID-19 are sustained in the longer-term. The structure of the article is as follows. First, the theoretical framework is outlined in some detail. Then, the qualitative study is described and the empirical findings are outlined. Next, the findings are discussed in light of the theoretical framework. The article concludes with commentary on the contributions made by the study for theorizing about health system change under stress.

## Understanding integrated health systems as complex, social and context-dependent

“*It is through relationships that an organization is able to make sense, learn, and improvise to manage the unpredictable trajectories of health*” [(17), p. 14].

Health systems are inherently complex (1, 14, 17); and this is in part because, like all other open social systems, they are comprised of people and (re)produced by human action (18). It has been argued that health systems are themselves “social constructions” [(19), p. 1] and “social institutions” [(20), p. 1463], that are “brought alive through the relationships among the actors involved in managing, delivering, and accessing health care” [(19), p. 2]. As such, it is critical that research and policy analysis recognize health systems as dynamic cultural, socio-political phenomena and not merely as “delivery points for bio-medical interventions” [(20), p. 1463].

Understanding healthcare as a system that is both complex and human-centered provides a promising frame for health reform research that seeks to address health disparities (17, 21, 22). This is because it allows us to draw on complexity concepts to both explain why the system operates in the way it does, but also how it (and us as agents) can be steered in a “more favorable direction” to ensure better access to universal integrated care [(14), p. 1].

Central to theorizing health reform in this way is the importance of context and relationships (i.e., inter-dependencies) and how these contribute to the process of “emergence” that impacts on health system functioning. Emergence here refers to “the arising of novel and coherent structures, patterns and properties during the process of self-organization in complex systems” [(23), p. 49]. In other words, emergent properties are the macro-level processes that occur in health systems due to the persistent interactions between system components via agents at the micro-level. That is to say, agent interactions combine together or act on each other to produce new processes, structures or components which are more than the sum of their parts. In the US, for example, [(14), p. 2] argues that the current fee-for-service system (context) discourages sharing of care responsibility between providers (self-organizing behavior via agent interactions) leading to reduced operational efficiency (an emergent property of a complex system).

Mitigating health system fragmentation by fostering effective communication, synergy and collaboration between and within organizations, sectors, teams and settings is paramount to developing accessible, universal, coordinated care systems (22, 24). Yet this process is complicated by the fact that integrated care is, in practice, “strongly context bound” [(25), p. 2]. Access to universal integrated care can therefore take different forms, require different facilitators and face different implementation challenges, depending on the existing health system and socio-political context in which it is being delivered (26). That is to say, a range of integrated care trajectories can develop that are evolutionary, historical and context-dependent (27).

In a complex (i.e. non-linear) health system of this kind that is sensitive to initial conditions (i.e., context) (17), new integrated care trajectories, then, are formed only when an enabling environment is created in and sustained by a health system over time. Such system conditions are generated when a specific mix of:

1. Strategies (actions enacted individually or collectively by health professionals);
2. Implementation mechanisms (processes or events through which strategies can be operationalized to achieve desired outcomes); and
3. Contexts (both internal and external to the health system).

Come together in a way that effectively connects a network of multidisciplinary, multisectoral and inter-organizational professionals to facilitate the provision of accessible, coordinated care (28, 29). In other words, it is a collective process and although working together, these actors may have different views, interests and objectives (30). For this reason, as Zonneveld et al. point out, “deeper understanding of collaboration and behavior in integrated care is needed” [(26), p. 2].

## Linking the micro, meso, and macro levels: Functional and normative integration

Since integrated care links primary and acute functions “by using a team-based approach to address the needs of the whole person” [(31), p. 2], health systems form a dynamic web of human interactions where collaborative and joined-up thinking are critical to both patient/provider wellbeing and system performance. Yet, enabling relationship-building, cooperation and coordination processes that connect different parties across acute and community care settings is a complex process that requires “time, interaction, and focused attention” [(32), p. 231].

From a complexity perspective, we know that health systems operate on the micro (clinical), meso (professional and organizational) and macro (system) level. Because of this, understanding of the key types of whole-of-system integration that ensure connectivity between all system layers is critical to research on the development of enhanced community care and new integrated care trajectories in the COVID-19 context.

Drawing on the work of (33), we therefore focus in this study on: (1) functional integration i.e., key support functions and activities to coordinate and support accountability and decision-making between agents (e.g., financial, management and information systems); and (2) normative integration i.e., the development and maintenance of a common frame of reference between agents such as shared mission, vision, trust, values and culture.

Indeed health systems research and analysis from Ireland, the UK and the US - undertaken either prior to or in some cases following the onset of COVID-19 - has signaled that the presence or absence of features linked to functional and normative integration can significantly influence the extent to which collective or coordinated action is facilitated or not (4, 12, 20, 25, 31, 34).

Notably, a Delphi Study conducted in The Netherlands reported that features linked to functional integration were viewed as less appropriate for health system functioning by experts, while soft enabling or normative features (including those linked to collective attitude, reliable behavior, conflict management, shared vision, trust, linking cultures and visionary leadership) were considered to play “a crucial role in the development of various complex inter-sectorial, inter-organizational and inter-professional service models of integration” [(29), p. 10].

## Research aims and objectives

This study forms one work package within the Health Research Board (HRB)-funded Foundations' applied research project that aims to harness key learnings from Ireland's health system response to COVID-19, with a view to informing the implementation of Ireland's ten-year health reform plan: Sláintecare (6, 35–39).

In its broadest terms, this “arm” of the research aims to advance understanding of how and to what extent COVID-19 has highlighted opportunities for change that impacted on access to universal integrated care in the Irish health system. More specifically, we set out to:

1. Generate in-depth insights into how and why particular health system responses emerged, scaled or pivoted during COVID-19;
2. Identify key strategies, implementation mechanisms and contexts that enabled or hindered better access to universal integrated care during COVID-19; and
3. Discuss key learnings for Ireland and internationally for health system reform in the COVID-19 context.

Applying complex systems thinking directly to the empirical, primary data described above, this study generates evidence that can steer health reform via strengthening public health and primary care functions while also tackling health inequalities. A common critique of complex systems theory in the field of health is that it is based largely on abstract discussions and is metaphorical in nature (21). By examining health system elements and effects that have been the subject of prior theorizing but not of prior empirical study, we provide important insights from Ireland's pandemic response that shed light on how we might better disentangle, understand and find novel solutions to implementing effective health system change in the longer-term.

## Methodology

### Study design

Complex systems research in the health field typically requires approaches and methods that are “situated in the qualitative paradigm” [(17), p. 6]. This is because in the complexity worldview, the non-linear, dynamic, co-adaptive and emergent character of social systems means that “we can never establish general non-contextual laws” [(40), p. 2]. From this perspective, quantitative approaches analyzing relationships between discrete variables are limited since they cannot explain how or why a health system trajectory, for example, changes from one state (unintegrated) to another (integrated) (18). Further, it was proposed earlier that health systems are inherently human-centered and socially constructed since they are derived “through human behavior and interpretation, rather than existing independently of them” [(19), p. 2].

As such, this study adopted a qualitative approach to explore Irish health professionals' experiences and interpretations of social processes of change that enabled or hindered better access to universal integrated care during the pandemic (41). Rather than seeking generalization, the use of open-ended questions facilitated the production of contextualized and in-depth insights into how (and why) specific circumstances and events impacted access to integrated care following the onset of COVID-19.

Unlike quantitative methods that necessarily decontextualize data to generate testable variables, qualitative methods employ a whole-person and dynamic perspective that situates individuals in their real-world settings (42). A nuanced and complexity-sensitive approach of this kind is critical to health systems research since, as [(43), p. 45] reminds us:

From one person we can recover social processes and social structure, networks, social change and so forth, for people are located in a social and cultural environment which constitutes and shapes not only what we see, but also how we see.

### Sampling and recruitment

The purpose of this study was not to generalize but to produce thick context-specific descriptions that provide explanatory insights into the processes that influence access to integrated care following the onset of COVID-19 in Ireland (44). As such, fewer cases were preferred to facilitate intensive engagement as well as deep case-analysis within the time available (45). Equally, it was important to ensure that the qualitative sample was not so small as to preclude the telling of a rich story, often referred to as informational redundancy (46). In keeping with the recommendations of Braun and Clarke (47), who suggest 10–20 participants to facilitate thematic analysis in medium-sized research projects, a total sample size of 16 health professionals was therefore considered sufficient to identify themes across the data.

Inclusion criteria for the study determined that those eligible to participate were frontline health professionals or senior health system managers from either acute and community settings, who were involved with one or more health system responses that: (1) pivoted, scaled up or emerged following the onset of COVID-19; and (2) could provide important insights into universal access to integrated care. This approach was underpinned by the belief that these health professionals were experts with specialist knowledge on the topic given their lived experience of working in and with the health system to provide access to integrated care during COVID-19.

Purposive sampling techniques (48) were employed to ensure diversity of experience across the sample in terms of context, system/seniority levels, settings (i.e., acute vs. community) and outcomes (i.e., responses that experienced both successes and significant challenges in providing better access to integrated care). As part of the parent study's co-production approach (35), the research team liaised extensively with the Project Steering Group including partners in the Health Service Executive (HSE) and Department of Health to identify a range of bottom-up^1^ (*n* = 7) and top-down^2^ (*n* = 4) responses to use as recruitment sites. This process began in April 2020 and, following a rigorous short-listing process where the most relevant responses were selected, resulted in the inclusion of GP Access to Diagnostics, the national vaccination roll-out, Chronic Disease Management programmes and Sláintecare Healthy Community programmes as well as initiatives in, for example, unscheduled acute and cardiac rehab care.

Table 1 presents background/summary data of the sample, broken down by gender, in terms of their role and function in the health system as well as the type of organization from which they were recruited. Amongst the sample as a whole, estimated years of experience working in the health system included 10+ (*n* = 1), 15+ (*n* = 6), 20+ (*n* = 5) and 25+ (*n* = 4).

**Table 1:** Sample profile.

**Sample background/summary data**	**Female**	**Male**	**Total**
**Health system workers recruited from bottom-up responses**	**5**	**4**	**9**
**Charitable organization**			
Management/admin	1	0	1
Psychology	1	0	1
**HSE**			
Consultant	1	2	3
Nursing	2	0	2
Occupational therapist	0	1	1
**Private company**			
Management/admin	0	1	116.5pt
**Health system managers recruited from top-down responses**	**4**	**3**	**7**
**HSE**			
GP	0	1	1
Management/admin	1	2	3
Occupational therapist	1	0	1
Physiotherapy	1	0	1
Public health physician	1	0	116.1pt
**Grand total**	**9**	**7**	**16**

### Data collection and analysis

Semi-structured interviews were the study's core method of data collection. Acting as a conversation with purpose (49), this method provided a means by which to thoroughly explore health professionals' experiences and perspectives by allowing for elaboration of topics deemed personally significant, while also ensuring that the major and most relevant topics were covered (50). Qualitative interviewing can pose challenges related to recall and selective memories; however, these issues are tempered since qualitative research is not concerned with the positivistic search for objective facts. Rather, it is considered both valuable and valid “for the express purpose of understanding people's interpretations of their world” [(51), p. 9].

Following ethical approval from the Research Ethics Committee of the Centre for Health Policy and Management and Center for Global Health in Trinity College Dublin's School of Medicine, data collection took place over a three-month period between March and May 2022. The interviews took place via online video conferencing and due to the understandably busy schedules of participating health professionals, ranged between 36 and 75 minutes, with most lasting between 45 and 60 minutes. The interview schedule covered a range of topics, including the background and triggers for the response, the impact of COVID-19 on its development or direction, facilitators and barriers to implementation and key learnings or reflections on enabling better access to universal integrated care during a crisis (see Supplementary material for more detail).

With participants' consent, all interviews were digitally recorded and transcribed verbatim (assisted by otter.ai).^3^ Adopting a team-based approach for applied researchers, we used word processing and spreadsheet software (Microsoft Word and Excel) via an online document management and collaboration platform to structure and organize the data for analysis (52, 53). Two researchers (SP and LMC) analyzed (i.e., coded) the data, while a third member of the team (SB) coded ~10–20% of same. Following numerous in-depth team discussions, this culminated in the development of coding categories related to themes and conceptual constructs that were emergent and grounded in the data rather than developed a priori (54). The coding process meant that data related to a range of specific topics could be extracted from each participant's narrative and collated into corresponding codebooks or files (55).

Salient patterns and observations were teased out through an in-depth analysis of the data in each topic-specific codebook, which facilitated the interrogation of key concepts and themes (56). Adopting a complexity-informed approach, dedicated analytic attention was also paid to the interactions between different components of the health system via agents to help explain the patterns observed (57).

Although complexity had been identified as potentially relevant to the analytic approach prior to data collection, an inductive approach to theorizing was used throughout the analysis stages of the research. Theorizing, in the social sciences, refers to attempts to understand or explain phenomena and is distinct from theory, which is the final or fixed articulation. In this study, transcripts were analyzed for themes and concepts relevant to answering the empirical research questions outlined above. During this process, the research team regularly discussed the continued relevance of complexity in light of the emerging patterns of observation. This led to the identification of emergence, inter-dependedness and self-organization as key principles to be utilized and ensured that the conceptual framework employed was ultimately driven by the findings.

In keeping with recommended practice and procedures for qualitative analysis, a number of measures were taken to ensure the trustworthiness and credibility of the interpretation of data (58). These included checking data for negative cases (i.e., outliers) (59) and regular discussions between the researchers that enabled multiple perspectives, insights, and interpretations to be considered (60). The analytic approach was also guided by the perspective that saturation was achieved when no new information on dimensions of experience or meaning were emerging from the data (61).

## Findings

We present three themes developed through in-depth interrogation of the data. Following this, we use a complexity lens to discuss key implications for health reform in Ireland and internationally.

*Theme 1: The pandemic response fostered opportunities for integration by providing a shared goal that helped to break down boundaries between previously fragmented care sectors, settings and cultures*.

Effective responses to COVID-19 required quick, collaborative and large-scale actions. While several participants noted challenges related to redeployment in the community sector, most spoke repeatedly about how the pandemic brought diverse teams and organizations across acute and community settings together, often for the first time, to provide better access to integrated care: “*I think that the very notion of the integration is, is probably the biggest shift”* (Health System Worker 2); “*COVID has taught us people don't want to be going in there [hospital]. So I think it's wonderful, the concept of integrated care. We've all been in our silos for years”* (Health System Worker 1). In fact, many discussed how, prior to COVID-19, they did not “*know about*” or “*fully understand*” other sectors or organizations in terms of how they worked or the structures that underpinned them, while a smaller number noted a history of mistrust and lack of information-sharing between, for example, the public and private sector.

Yet, during the pandemic, participants said that health professionals “*just threw off the labels*” and developed a “*we're all in this together*” perspective to enable effective collaboration of their shared purpose: providing effective and universal coordinated care during a crisis. Through repeated interactions between agents across different parts of the health system that would have previously had little contact, the pandemic response thus facilitated the development of what participants often described as a joint awareness of each others role in the health system as-a-whole.

Critically, this more nuanced, co-produced and macro-level understanding of the health system: (1) led to knowledge-generation about existing gaps and how the different parts of the system could work better together to address them; (2) empowered and energized health professionals by showing them that health system reform via integrated care structures was possible; and (3) challenged long-standing cultural mindsets by engendering a strong appreciation of the need for and value of, community services in providing better access to universal integrated care:

“*I couldn't see the gaps before, not until you're in it. So it's helped us kind of see where we could be more supportive to the community, but also how we can improve the interactions of community-based services with the unscheduled care system*.” (Health System Worker 3)

“*I suppose, for me, it reaffirmed my faith in the people working in the system, because we said, ‘Look, we're focused on the patient here'. What's encouraging is that people talk about person-centered care, but this was a real manifestation of it.”* (Health System Worker 1)

“*When COVID hit we were only bringing in the sickest of the sick. Whereas before, there definitely would have been a mindset among people working in the acute environment, that ‘Oh, no, everybody has to come into us we'll see them in clinic'. So that's definitely the shift in mindset that, you know, we [in the community] can look after them now. It doesn't work for all patients. But it certainly worked well in this particular project.”* (Health System Worker 8)

COVID-19 therefore not only validated the need for agents across all components of the health system to work together, but also tangibly demonstrated the value of doing so, if only temporarily. Indeed, many spoke about how they felt the “*momentum*” generated through the pandemic response was already lessening and expressed strong concerns about a return to traditional silos of care post-COVID-19: “*If I'm honest, I'm concerned that when the light dims on the [community] sector, people will start going back into their old ways of doing business. That is a real concern, from my perspective*” (Health System Worker 8).

Health professionals discussed how embedding a complex or interdependent understanding of the health system would also help to mitigate many issues that can hamper the goal of achieving better access to universal integrated care. For instance, some talked about how their response did not fit neatly into the category of acute or community and felt they would have benefited from clarity in terms of where they fall under current governance structures during the pandemic, while, on the day-to-day side of things Health System Manager 1 summed up the importance of whole-of-system visibility for integration by saying: “*you' can't send a patient to services that you don't know exists, or you don't know how to access”*, reiterating that “*the key to unlocking the door to a referral pathway is knowing who's the person that you talk to”*.

Several also emphasized how greater awareness of the interdependent nature of the health system could help prevent overreliance on particular responses or sectors. For instance, Health System Manager 3 talked about how an unscheduled care initiative was so impactful in terms of hospital avoidance during COVID-19 that it became a victim of its own success, noting that: “*yes the [response] is good, it has a place, but it's not a panacea”*. In other words, no one response, service or sector should be viewed as a magic bullet; rather, better access to universal integrated care will require agents to collaborate effectively across care settings and disciplines to build a more connected health system. As Health System Worker 7 put it:

“*[COVID led to the realization that] the acute hospital is more than its walls, that you can't be limited by the buildings of an institution in what you do. And I do think the whole hospital is much more attuned to that now. And that's the biggest reform, I think, the use of increased community-based services.”*

Finally, the narratives revealed how clarity from leadership about commitment to universalism – a core plank of which is integration - was necessary to maintain the shared goal of a fairer system that was mobilized during COVID-19, as was the need to communicate this message effectively:

“*I think clarity from the system around our commitment to the universal piece is probably important. We've got a taste for it now [referring to the universal nature of the pandemic response] it's created a fairer health system. And I think that's an important thing to people; that they feel this system is fair. But are we serious? Are we really committed to that? Hopefully that's a value that we can keep hold of and people will continue to buy into.”* (Health System Manager 7)

“*We struggled to communicate down our system in a cohesive way [during the pandemic]. There's different levels of our system - some understand, some don't and some don't know or are just learning. So how you translate something and engage people becomes very significant.”* (Health System Manager 6)

*Theme 2: The pandemic response created system conditions that enabled innovations to foster integration; yet, funding (and other) structures to maintain these solutions in the longer-term remain unclear*.

Many health professionals talked about how the pandemic forced them to think outside-the-box in developing new ways of working or providing care: “*[COVID showed us] that you can no longer think that the service can only be delivered one way, you have to think of other ways*” (Health System Worker 3). A majority of these strategies involved telemedicine, access to resources and technology and flexibility, adaptability and the use of virtual platforms to facilitate communication channels between multi-disciplinary teams (MDTs). However, a core overarching theme was a shift in focus toward patient wants and needs – i.e., moving services from hospital closer to home - rather than simply managing an institution as a place of care: “*We realized a lot of our models just weren't fit for purpose because they were face-to-face, so we had to adapt”* (Health System Worker 7). Equally important was the system- and organizational- level modifications – such as changes in procurement processes and procedures - that *enabled* innovation and rapid change in direct response to the crisis. As one health system worker explained:

“*[COVID] allowed stuff to progress much more quickly than it would otherwise have done, because it circumvented a lot of those institutional barriers … anything we thought would improve and innovate was facilitated, and they've been proven to be correct. Whether it was equipment, whether it was small infrastructural issues, whether it was staff, you know, and it really did change it.”* (Health System Worker 4)

In other words, the open and flexible system conditions created in and by the pandemic response meant that health professionals felt encouraged (and supported) to not only develop solutions that were effective and responsive to their community's needs, but to also figure out what worked and importantly, what did not and why. In fact, several spoke about how innovation flourished since it was largely facilitated by a hands-off top-down approach, where the health system provided funding and other necessary structural supports, but then “*let the frontline get on with it*” in responding to the crisis. Yet, a number observed that such system conditions were already starting to show signs of reverting to type, with one participant noting that “*now we're back to budgets, adherence, staff cuts. The system is like ‘You've got to watch your WTEs. What's your agency spend? What's your overtime spend?' It's just revert to type”* (Health System Worker 4).

Moreover, while health professionals agreed that the pandemic response gave them “*permission to be innovative*”, they often described funding models – including those that existed pre-COVID-19 - as posing challenges since they tended not to be prospective and/or long-term in nature. For instance, several health system workers spoke about the challenges associated with funding drops that were often unexpected and politically charged, therefore fostering competition and hasty planning rather than iterative and sustainable solutions. As Health System Worker 5 from a bottom-up response put it: “*We got 2 weeks' notice there's suddenly money, we suddenly have to spend it and therefore we put in these projects that could have been done a lot better and planned a lot better if you ask me”*. Likewise, this problem also manifested in the top-down responses and was often linked to a lack of certainty in terms of multi-year funding. As Health System Manager 2 described, their current funding model led to job insecurity for their staff, which ultimately undermined the effectiveness of the response: “*The Programme is run by peer leaders that we train. It works much better that way. But because of the way we fund, there is a huge risk around continuously losing people*.

Health professionals also pointed to the importance of both creating and embedding mechanisms to share information in a systematic way, so that proven models and innovation from the pandemic response can be adapted across different settings and organizations where appropriate. Several participants talked about how this kind of information was not currently or sufficiently being communicated *to* the system *by* the system. As a consequence, healthcare workers were devising new ideas and business plans from scratch, rather than building on what was already there, sometimes leading to additional stress and burn-out. In the following quotes, a health system worker reflected on how this approach was perceived as neither effective nor efficient for fostering integration in the longer-term, while a health system manager reiterated the importance of documenting pandemic innovations of this kind so that they can be fed directly back into the system to bolster health system preparedness:

“*We don't need to be reinventing the wheel all over the country; just look at examples of good innovation and good integration and try and replicate that … It's only by me sourcing it or seeing it on Twitter when I say ‘jeez, I could do that'. And that's where I get a lot of my ideas, but it's not the system telling me.”* (Health System Worker 3)

“*We need to be looking at multiple elements - the ICT [information and communication technology], the workforce, the procurement, the logistics - so that you're not going back to scratching your head if another pandemic happens … lesson number one is that intelligence is documented so you'll never be back at* zero.” (Health System Manager 1)

*Theme 3: The pandemic response highlighted the importance of relationship-building and trust in facilitating effective collaboration to improve universal access to integrated care*.

Interpersonal relationships and relational efficacy were frequently described as equally if not more important than practical enablers (such as ICT and procurement processes) among those working on the frontline and at a more senior managerial level in the health system during the pandemic: “*Far and beyond technical issues, it's people coming together and actually seeing that it works and that there are benefits to them that made the biggest difference”* (Health System Manager 6). This largely stemmed from the belief that you can have all the right procedures and structures in place for integration, but without collaborative relationships they will not be effective because the system is ultimately made up of and run by people who must work together to implement change.

In fact, participating health professionals framed almost all system interactions as relational, with some emphasizing how informal networks can sometimes be just as influential as formal ones when it comes to information-sharing and decision-making: “*It's a very human thing … you can be sure that various people [in the health system]pick up their phone to talk to their buddy [to gain clarity on certain issues] and that's very understandable*” (Health System Manager 1). And while the findings presented in Theme 1 highlighted the importance of increased contact between diverse settings and sectors to enable better access to universal integrated care during COVID-19, what was perceived as equally critical by participants was the nature and quality of those interactions.

For instance, many health professionals observed that during the pandemic, communication between different sectors, settings and organizations was greatly improved in that it was regular, ad hoc and conducive to immediate problem-solving. For example, several spoke about how they were picking up the phone to ring senior health managers directly when issues arose, while others were having frequent meetings with wider MDT teams that would not have met prior to COVID-19. Participants explained that engagements such as these helped to build a level of trust that facilitated cooperation and coordination between different system levels that enabled better access to integrated care in the midst of the crisis that will hopefully continue post-COVID-19. As Health System Worker 4, from a nursing home response team, explained:

“*We've a weekly meeting, which has gone to two weekly with public health and the local care area. That started out in COVID and it's been really good, because we still meet regularly and now we're talking more about monkeypox and things like that, and the implications for the system. So that link has been so useful, because we've all developed this whole kind of, you know, we all trust each other, we all understand what we're trying to do.”*

Others reflected on the importance of sensitizing each other to organizational and cultural differences to ensure effective collaboration between integrated services, such as conflict management and communication styles: “*We didn't really have any understanding between the two organizations in terms of differences between how people managed conflict, how people managed things when they go wrong and things like that. [So] there was big learning there*” (Health System Worker 8). Just as importantly, the development of trust and strong relationships during COVID-19 bolstered buy-in and a belief that certain responses could and should work, which was ultimately seen as contributing to their success. As Health System Manager 6 put it: “*[COVID] showed us that if you have a model that people buy into and believe in, no matter how challenging, you'll get it done … and that's to do with winning hearts and minds*”. However, as was noted numerous times amongst participants, the goal of winning hearts and minds was not something that happened by chance; rather, as Health System Manager 6 reiterated: “*It takes constant work … it's about building capacity and capability [in the system] to actually engage, negotiate and plan a strategy [to facilitate trust- and relationship-building] in a programmatic way”*.

What emerged strongly from the narratives was the role of honesty in this process and, more specifically, the need to build a culture of honesty across all system levels to facilitate effective collaboration, problem-solving and sustainable solutions. In the following quotes, a health system worker from a bottom-up response and a health system manager from a top-down response both reflect on how honesty was critical for conflict management between different organizations working together to provide better access to integrated care during the pandemic:

“*The relationships from the start were really good and have remained so. And that was because of the tone set by a couple of the senior people involved … there was a huge degree of trust needed and honesty is linked to trust and there was an honesty on both sides … for example, there was an expectation around a piece of funding that didn't arrive but we got over that, because there was an honesty there.”* (Health System Worker 8)

“*We used the process of negotiation to build the relationships and we started to get to the place of a fair and honest engagement, where trust was built across the table, but also it wasn't all one sided … Doesn't mean that we don't have significant disagreements, but when the relationships are solid, we get through them.”* (Health System Manager 7)

Others, however - particularly those on the frontline - pointed to ongoing issues related to a perceived lack of transparency in leadership and engagement in decision-making that negatively impacted trust and relationship-building during COVID-19: “*I understand the structure [of the health system] and who's at the top, but it's never clear how exactly decisions get made”* (Health System Worker 9); “*Nobody sought any advice or opinion on how this particular project can be transitioned to [existing national programme]* (Health System Worker 1). This points to the need to build what one health system manager described as a “*coalition of support*” across all system levels - i.e., where leadership, organizations, the political system and frontline workers are engaged and brought to the table: “*During COVID, we developed relationship managers who manage the process with us. So that's an interesting innovation, which has to do with relationships”* (Health System Manager 6).

## Discussion

This research examined how and to what extent COVID-19 highlighted opportunities for change that enabled better access to universal integrated care in the Irish health system. A qualitative study was undertaken through interviews conducted with health system workers and managers directly involved in the pandemic response. Adopting a complexity-informed lens, we now interpret the findings by applying complexity concepts and principles to better understand how new integrated care trajectories emerged during COVID-19 and discuss the policy and practice implications for health reform. Three key learnings from the pandemic response are presented: (1) nurturing whole-system thinking through a clear, common goal and shared information base; (2) harnessing, sharing and supporting innovation; and (3) prioritizing trust and relationship-building in a social, human-centered health system.

### Nurturing whole-system thinking through a clear, common goal, and shared information base

While it is acknowledged that redeployment in the community sector posed challenges in some cases (39, 62), enabling better access to universal, integrated care during COVID-19 was nevertheless a complex process that took place at multiple levels across various interventions and involved numerous stakeholders and contextual nuances. The pandemic – which in complexity terms would be characterized as a “substantial perturbation of the system” [(18), p. 3] – engendered a shared goal amongst health professionals: to provide access to universal, holistic care in the midst of a crisis (13, 63). This, in turn, precipitated rapid and mutual adaption in the form of strategic efforts to foster emergent inter-organizational and cross-sector collaborations between previously disconnected “parts” of the system – a self-organizing process that Comfort et al describe as “coordination in practice” [(64), p. 64]. In this way, health professionals became “conscious of the system in which they reside” [(18), p. 3]; they demonstrated an awareness of the complexity or interdependent nature of healthcare by acknowledging that action (or inaction) in one part of the system had the potential to impact others in significant ways.

From this perspective, enabling better access to universal integrated care during COVID-19 involved inter-professional coordination that was largely a voluntary activity sustained by a clearly articulated and shared vision or purpose (64). The findings thus reiterate the power of creating (and embedding) a shared goal to drive change in complex (social) health systems that are sensitive to initial conditions. It is generally accepted that this process should involve a “participative and focused dialogue” among diverse stakeholders [(65), p. 99]; however, further research on what this unifying message should be outside of crisis periods and how it should be created (and communicated) in ways that take account of critical contextual factors, and how they interact and change over time, is needed (66).

Moreover, health professionals providing integrated care during the pandemic required timely, accurate and relevant information that empowered them to adapt their actions in response to changing conditions and shifting priorities (64). The findings suggest that an important route for reform in this area would be to mobilize collective action by nurturing a whole-of-system perspective (67). This could be achieved by developing an active, living map of the health system that clearly identifies (and regularly updates) key components, governance structures, services and access-points and is accessible both during and outside times of crisis.

In Ireland, this has been successfully achieved for some specific population groups and/or within certain clinical programmes [e.g., (34, 68)]. Yet a shared knowledge base that links the health system as-a-whole does not currently exist. Critically, such a tool would allow for the exploration of multiple potential solutions by improving system awareness, identifying interdependencies, providing clarity in terms of accountability and fostering inter-professional collaboration and learning (65); all of which would help to enable better access to universal, integrated care in the COVID-19 context and beyond.

### Harnessing, sharing, and supporting innovation

The pandemic response necessarily led to significant system change to allow for agile, speedy solutions to emerge in response to the crisis, primarily with regard to increased funding and the relaxing of procurement processes and fast-tracking of digital health responses (35). Traditional, formal structures and hierarchies were therefore removed which in turn, enabled “more horizontal collaboration” and decision-making that sparked innovation [(26), p. 3]. Innovation, then, was an emergent (macro) property of the health system (69) that occurred from the bottom-up as a result of agents interacting to facilitate shared sense-making, a process that is “fundamental to supporting adaptation” in complex systems [(18), p. 5]. In this way, the lifting of procedural barriers represented a small change or perturbation in system conditions (acceleration) that led to a significant or non-linear emergent effect (innovation) that occurred due to the self-organizing behavior of agents (1). Through these complex processes, uncertainty was harnessed into positive adaptation and innovative practices to enable better access to universal integrated care during COVID-19.

However, since innovation of this kind can be characterized as an emergent and evolutionary process that unfolded in an unpredictable and unplanned way, key learnings should be constantly refined, developed and fed back into the system to maintain their relevance and maximize their impact post-pandemic (70). Indeed, the findings indicate that the pandemic response created a space for out-of-the-box thinking or in some cases, an avenue through which to action previously and sometimes long-held ideas about how to enable better access to universal integrated care. This ensured that the system remained adaptive during the crisis by empowering health professionals through top-down support, encouragement and trust to build on their strengths, to engage in important trial and error solutions (viewing failures as opportunities for learning and improvement) and to generate a sense of ownership in decision-making (22, 26, 71). Yet the narratives revealed that the health system was already starting to revert to type by reinstating priorities and procedures that can potentially undermine the non-hierarchical collaboration, adaption and information-sharing necessary to develop and importantly expand novel solutions.

Systems theory teaches us that in situations where a low level of uncertainty exists with regard to problem-solving, standardization and traditional hierarchical structures are important and necessary to enhance efficiency (72). However, where a *higher* level of uncertainty exists – such as in response to complex challenges - leadership should consider tasks and approaches that are accomplished by emergent, relational dialogue among diverse health professionals (73). Both approaches can and should be able to theoretically co-exist in a health system, whereby: (1) adequate space, time and resources are provided to stimulate and curate innovation on the frontline to identify “sustainable solutions hidden within plain sight”; and (2) such innovations are then institutionalized through top-down (traditional) control mechanisms [(18), p. 5].

Health system change should thus recognize that social dynamics, reciprocal learning, effective communication processes and the promotion of exploration are all foundational to developing adaptive, innovative solutions (73). Perhaps, then, a critical learning for health system leadership and reform from the pandemic response is the importance of not only providing answers, but also asking questions (74).

### Prioritizing trust and relationship-building in a social, human-centered health system

Enabling better access to universal integrated care during the crisis meant that professionals across a diverse range of health sectors, settings and services had to work together and collaborate, often for the first time in the Irish context. Collaboration necessitated interaction; and all interactions that occur between humans operating in a complex social (health) system – whether formal or informal - are relational (20, 75). However, what emerged strongly from the findings of this study was that effective collective action during the pandemic went beyond physical, electronic or structural proximity within and across acute and community settings; rather, basic human connection, relationship-building/management and the development of trust were all considered fundamental enablers to coordination (22, 26, 76). Thus, health reform efforts to improve access to universal integrated care in the COVID-19 context should not only focus on integrating structures or improving individual components but should equally consider strengthening relationships among those working together across all system levels (17, 22).

Yet as Adam and Donelson point out, trust and other relational issues can be difficult to define and measure in the context of health system change since they lie in-between; “in-between people and people, in-between people and organizations, and in-between people and events” [(75), p. 119]. Nevertheless, research evidence points to several ways health systems can engender an environment (that is, initial conditions, to use the language of complexity) that enables the development of various sets of mutual, trusting relationships. This includes, for example, a paradigm shift that is translated into cultural norms and a shared narrative where healthcare is (re)framed as relational rather than transactional (71). Culture and leadership are interdependent, synergistic and co-developed (77); as such, the need for compassionate, inclusive and collective leadership is central to this process, particularly at a time when health professionals are experiencing fatigue and burn-out post-pandemic.

An approach to health reform of this kind aligns with the complexity perspective by reorienting attentiveness to the nature and interactions of health professionals (system agents) to determine how uncertainty can be harnessed into positive adaptation (18, 22). Complexity-inspired leaders foster collaborative relationships and shared goals while also embracing chaos and creating a space for people to express their dissent or frustration. This, in turn, promotes “shared sense-making, exploration of strategic options through action and learning from those actions” (74). Begun and Thygeson suggest that to encourage this kind of interconnectivity between health professionals, leadership must enable collective and transparent decision-making to allow all voices to be heard, whilst also ensuring that quality standards are met and adhered to (73). Equally, to facilitate respectful interactions and minimize possible communication breakdown, policy and practice decisions need to be co-produced, with a focus on “enhanced communication flow and perhaps more importantly enhanced understanding of the information communicated” [(78), p. 23–24].

## Conclusions

This work conceptualized health systems as social and complex, and applied complexity concepts to advance understanding of how (and why) integrated care trajectories emerged following the onset of COVID-19 in Ireland. In doing so, we emphasize the role of reflexivity in system functioning, where human perceptions and actions are framed as both the cause and consequence of system dynamics (18). It is acknowledged that health reform is further complicated by the fact that health systems are constantly evolving, changing and adapting, both to internal and external stimuli such as the current health crisis. Health systems are context-sensitive and context-dependent; however, variability and uncertainty (i.e., complexity) of this kind is arguably a sign of system health (13).

By opening an active dialogue between empiricism and explanation to better understand the processes of change that enabled universal access to coordinated care during the pandemic, we have strengthened the potential contribution of the findings for informing health reform in Ireland and internationally. Unlike traditional health system approaches to reform that aim to reduce uncertainty, the findings open up new ways of thinking about health system change by encouraging health system leaders and policy-makers to embrace complexity. This, in turn, can enable alternative approaches to transformation that allow for exploration of multiple potential solutions to facilitate better access to universal integrated care in the COVID-19 context and beyond.

## Strengths and limitations

Using an in-depth qualitative approach, this study draws attention to both the extent of health system change as well as the complex dynamics of health system change that occurred following the onset of COVID-19 in Ireland. The seismic impact of the pandemic was experienced by all health professionals worldwide; yet, understanding of what this change looked like at a country-level, as well as the implications for access to universal integrated care, has hitherto been underexplored in the research literature. By applying a complexity lens to the study findings, the insights and analysis presented in this article provide a useful foundation for discussion and debate amongst health policy-makers, leaders, planners and academics. What is important now, is drawing on these lessons from the pandemic response to inform universal health system reform in a way that makes such solutions pragmatic and sustainable in the longer-term.

Notwithstanding, this study's insights should be understood in light of its limitations. The research was both undertaken in and specifically examined the COVID-19 context. The processes of change that occurred during this time within the Irish health system were therefore unique since it was responding to an acute and unprecedented crisis. Nevertheless, the findings demonstrate that the individual, organizational and system level changes required for large-scale health system reform to enable better access to universal integrated care are indeed possible, even if only temporarily. Moreover, critical insights have been gleaned that have important policy and practice implications for the development and implementation of health reform both in Ireland and internationally, especially in countries that have adopted (or are in the process of transitioning to) a universal health system.

As stated earlier, generalization was not the purpose of this (or any other) qualitative study. However, it is acknowledged that this research was unable to include accounts from health professionals working across all health system responses active during the pandemic. As part of the co-production process, the researchers worked extensively with health system leaders and experts to identify the responses considered most relevant, with a specific focus on those that enabled (or sought to enable) better access to universal, integrated care during COVID-19. Following this, and in keeping with the nature and rationale of the broader study within which this study is situated, the sampling approach prioritized diversity of experience and convenience to produce research evidence at speed that can be fed directly into the health system in real-time to inform health system change.

Finally, this study's findings are based on data from Ireland and cannot, therefore, be assumed to be applicable or transferable elsewhere due to contextual differences. Nevertheless, since relatively similar pandemic experiences have been, and continue to be, found across the developed world, it is reasonable to suggest that corresponding integrated care trajectories may well emerge in other countries. To this end, comparative studies may be a fruitful avenue for further research that aims to fully interrogate the contexts, strategies and mechanisms that influence the social processes of change necessary to drive health reform and enable better access to universal, integrated care in the COVID-19 context.

## Data availability statement

The datasets presented in this article are not readily available because due to the nature of this research, participants of this study did not agree for their data to be shared publicly, so supporting data is not available. Requests to access the datasets should be directed to SB, burkes17@tcd.ie.

## Ethics statement

The studies involving human participants were reviewed and approved by the Research Ethics Committee of the Center for Health Policy and Management and Center for Global Health in Trinity College Dublin's School of Medicine. The patients/participants provided their written informed consent to participate in this study.

## Author contributions

SP conceptualized the study design and methodological approach, collected the data, conceived the analytical, theoretical approach, performed the analysis, and prepared the original manuscript. LM conceptualized the study design, methodological approach, collected the data, and reviewed and edited the manuscript. RS reviewed and edited the manuscript. SB conceptualized the study design, methodological approach, acquired the funding, supervised the data collection, contributed to data analysis, and reviewed and edited the manuscript. All authors contributed to the article and approved the submitted version.

## Footnotes

^1^Bottom-up responses refers to those which emanated from the frontline, often from professionals providing on-the-ground care/services, who developed and implemented new and innovative ways of providing care to the public.

^2^Top-down responses refers to national responses which came from central Government/HSE and are implemented via a policy instrument (legislation, funding, regulation, guidance).

^3^While the AI technology utilised provided relatively accurate transcriptions, some inconsistencies were present. For this reason, the transcripts were revisited and cleaned by the interviewing member of the research team. On the whole, this resulted in a process that was significantly less time consuming than transcribing by hand.
