# Supplementary material for: How to not revert to type: Complexity-informed learnings from the pandemic response for health system reform and universal access to integrated care
Source: Front Public Health. 2023 Feb 17;11:1088728. doi: 10.3389/fpubh.2023.1088728 (PMC9996344; doi:10.3389/fpubh.2023.1088728)
Supplement: Supplementary file 1 [file Data_Sheet_1.PDF]

INTERVIEW SCHEDULE

Background info

1. Could you very briefly describe:
   - Your role in ______________
   - What triggered/drove the need for ______________?
   - What influenced the development of ______________?
2. Can you tell me in what way COVID-19 has influenced ______________? (prompt re development, direction and/or implementation).
3. Can you tell me about any shifts in thinking, if any, that you've noticed since the onset COVID-19? Either amongst yourself, your colleagues or from within the broader system?

Moving on from background, we're interested in learning about the key facilitators and barriers to creating better access to universal integrated care…

4. What do you and your colleagues do that makes this response work/successful?
   - What about at the service-level, collectively as a team?
   - And what about on an inter-service level?
   - In terms of what you just described, why, in your opinion, did this work?
5. What would you say is the single biggest thing that has contributed to this response's success? Can you say in one word.
6. What would you say are/were the main barriers to the successful implementation of ______________?
7. In what ways, if any,
   - do you feel the system has helped/supported of ___________to achieve its goals?
   - do you feel the system could do more to help ______________ achieve its goals in the short term?
   - You mentioned earlier that XXX was crucial for _________'s success, how do you think the system could help to support this, specifically?
8. What would need to happen to ensure that ___________continues to improve access to integrated care in the longer-term?

Key lessons/reflections on delivering integrated care in the COVID context

9. What, if any, are the impacts ___________has on future healthcare reform in Ireland as outlined in Slaintecare?
10. Knowing what you know now, if you could start/design/roll out ___________from scratch is there anything you would suggest be changed or done differently to ensure greater success?
11. Do you think being involved in ___________during COVID-19 has changed your perspective about the Irish Health system in any way? What about any of your colleagues?

Wrap-up

12. Is there anything you'd like to add that you didn't get a chance to say?
13. Are there any other questions/topics that you think would be important for us to ask about during this research?
